# Transient process of cortical activity during Necker cube perception: from local clusters to global synchrony

**DOI:** 10.1186/1753-4631-4-S1-S7

**Published:** 2010-06-03

**Authors:** Daisuke Shimaoka, Keiichi Kitajo, Kunihiko Kaneko, Yoko Yamaguchi

**Affiliations:** 1 Department of Basic Science, Graduate School of Arts and Sciences, The University of Tokyo, 3-8-1 Komaba, Meguro-ku, Tokyo 153-8902, Japan; 2 RIKEN Brain Science Institute, 2-1 Hirosawa, Wako, Saitama 351-0198 Japan; 3 PRESTO, Japan Science and Technology Agency, 4-1-8 Honcho, Kawaguchi, Saitama 332-0012, Japan; 4 ERATO Complex Systems Biology Project, Japan Science and Technology Agency, 3-8-1 Komaba, Meguro-ku, Tokyo 153-8902, Japan

## Abstract

**Background:**

It has been discussed that neural phase-synchrony across distant cortical areas (or global phase-synchrony) was correlated with various aspects of consciousness. The generating process of the synchrony, however, remains largely unknown. As a first step, we investigate transient process of global phase-synchrony, focusing on phase-synchronized clusters. We hypothesize that the phase-synchronized clusters are dynamically organized before global synchrony and clustering patterns depend on perceptual conditions.

**Methods:**

In an EEG study, Kitajo reported that phase-synchrony across distant cortical areas was selectively enhanced by top-down attention around 4 Hz in Necker cube perception. Here, we further analyzed the phase-synchronized clusters using hierarchical clustering which sequentially binds up the nearest electrodes based on similarity of phase locking between the cortical signals. First, we classified dominant components of the phase-synchronized clusters over time. We then investigated how the phase-synchronized clusters change with time, focusing on their size and spatial structure.

**Results:**

Phase-locked clusters organized a stable spatial pattern common to the perceptual conditions. In addition, the phase-locked clusters were modulated transiently depending on the perceptual conditions and the time from the perceptual switch. When top-down attention succeeded in switching perception as subjects intended, independent clusters at frontal and occipital areas grew to connect with each other around the time of the perceptual switch. However, the clusters in the occipital and left parietal areas remained divided when top-down attention failed in switching perception. When no primary biases exist, the cluster in the occipital area grew to its maximum at the time of the perceptual switch within the occipital area.

**Conclusions:**

Our study confirmed the existence of stable phase-synchronized clusters. Furthermore, these clusters were transiently connected with each other. The connecting pattern depended on subjects’ internal states. These results suggest that subjects’ attentional states are associated with distinct spatio-temporal patterns of the phase-locked clusters.

## Background

It has been discussed that phase-synchrony across distant cortical areas (or global phase-synchrony) was correlated with various aspects of consciousness [1-3]. However, it remains largely unknown what function such synchronous activities play in conscious processing. Recently, some researchers postulate that synchronous activity is not sufficient for consciousness, but assists enhancing a nascent assembly of neurons in its competition with other assemblies [1,4-6]. To clarify the role of transient global-phase-synchrony, it is crucial to reveal not only synchronous phenomena, but also the dynamical mechanism underlying the phenomena.

As a first step in this clarification, we focus on characterizing the transient process of formation of the global phase-synchrony. One possibility to form a global synchrony is growth of one small “core”. Another possibility could be the merging of coexisting clusters. These processes may depend on perceptual states such as consciousness and attention that accompany the global synchrony. As an example to investigate the transient process towards the global synchrony, we choose to analyze the global phase-synchrony around 4 Hz in Necker cube perception. In this system, phase-synchrony of electroencephalography (EEG) signals across distant cortical areas was selectively enhanced by top-down attention [7].

In this report, we analyze the spatio-temporal dynamics of phase-synchronized (or phase-locked) clusters using a hierarchical clustering algorithm. Hierarchical clustering binds cortical areas by linking pairs of cortical areas that are close in phase. With this algorithm, we first extract the dominant component of the clusters over time. We subsequently investigate temporal modulation of the clusters, focusing on their size and spatial structure. We thereby characterize the transient process of the attention-enhanced global phase-synchrony.

## Methods

### Subjects

Sixteen healthy human subjects with normal or corrected-to-normal vision (mean age: 24.7 years; SD: 4.7 years) participated in the study. The study was approved by the RIKEN research ethics committee, and the experiment was undertaken with written informed consent.

### Experimental task

**Figure 1:**
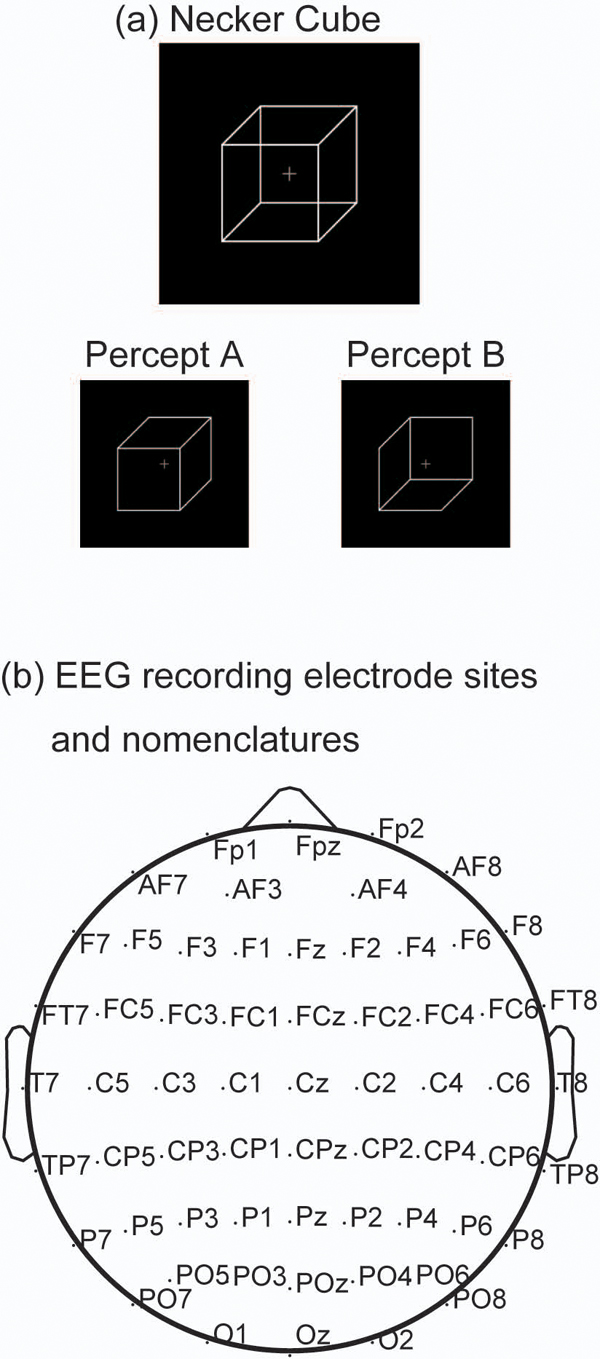
Visual stimulus and EEG recording sites

The subjects were presented with the Necker cube (width: 4.22°) on a black background at the centre of a 19 inch CRT monitor (Figure 1(a)). They were instructed to maintain fixation at a centre fixation cross on the monitor throughout each 180 s block and to avoid making eye movements and eye blinks. When one looks at the Necker cube, his/her perception switches between percept A and B (Figure 1(a)). During the 180 s period, the subjects reported switches in their perception by pressing different keys. In addition, they were instructed to follow one of the four perceptual conditions:

(1) Passive view condition (Neutral condition), in which they looked at the cube passively.

(2) Percept A biasing condition (Top down biasing condition 1), in which they tried to maintain percept A as long as possible.

(3) Percept B biasing condition (Top down biasing condition 2), in which they tried to maintain percept B as long as possible.

(4) Self-paced key pressing condition (Control condition), in which the cube was not presented and subjects pressed the keys at their own paces.

In addition to the above conditions, we set the base line condition (referred to as the Resting state condition) in which subjects looked at the centre cross on the blank screen without pressing keys.

In this paper, we will show the results for the switches from Percept B to A. These perceptual switches are classified into the following 3 cases. In the “desired” switch, they try to perceive A, and their percept switches to A. In the “undesired” switch, their percept switches to A, even though they try to continue perceiving B. In the neutral switch, they have no primary biases before the switch.

### Effects of top-down attention assessed with behavioural data

Behavioural effects of top-down attention were assessed with selective modulation of dominance durations. In condition (2), the dominance duration of percept A (desired percept) was significantly longer (Mann-Whitney U test, p < 5.00×10^-199^) than that of percept B (undesired percept). Likewise in condition (3), the dominance duration of percept B (desired perception) was significantly longer (Mann-Whitney U test, p < 5.00×10^-125^) than that of percept A (undesired perception). These results were consistent with a previous study [8]. We thereby confirmed that the subjects actually viewed the cube with top-down attention in condition (2) and (3).

### Electroencephalographic recordings

Cortical activity was recorded using a 62 ch EEG (Figure 1(b)). Electrooculogram (EOG) was simultaneously recorded at the position 1 cm from the outer canthi of both eyes and above and below the left eye. EEG and EOG were amplified with a gain of 500 (Neuroscan, EL Paso, TX), band-pass filtered between 0.1 and 100 Hz, and digitalized at 500 Hz. Scalp voltages were referenced to a linked earlobe reference. Electrode impedances were kept 5 kΩ. One EEG epoch was defined as a period between 1.5 s before and 1.5 s after a key press that reported a perceptual switch. Epochs with artefacts caused by blinks or eye movements or amplifier saturation were detected using an amplitude criterion (±100 μV) and excluded from further analysis. EEG epochs were extracted in which perception of the rivalling views persisted for 1 s or longer both before and after the perceptual switching. A total of 398 epochs for each perceptual condition were obtained from 16 subjects. To reduce the effect of volume conduction, EEG scalp current density [9] was calculated from the raw signals. For a visualization of topographic maps, EEGLAB Matlab toolbox was used [10].

### Data analysis

####  Detection of phase-locking

Scalp current density data were band-pass filtered around 4 Hz (3.6 – 4.4 Hz). This frequency was chosen because average phase-locking across recording electrodes was higher at this frequency than at other frequencies [7]. The instantaneous phase was extracted from the analytic signal calculated with the Hilbert transform. Degrees of phase locking between signals recorded from different electrodes were measured as Phase Locking Value (PLV) [11]. PLV quantifies how consistent the phase relationships between the two signals are across epochs at a given time. PLV between signals from electrode *i* and *j* at time *t* is given by:

where *N* (= 398 for each condition) is the number of total epochs and *ϕ*(*t, n*) represents the instantaneous phase at time *t* in the *n-*th epoch. PLV ranges from 0 (random phase difference or no phase locking) to 1 (constant phase difference or perfect phase-locking).

#### Relative change of phase-locking

To extract the relative change of phase-locking from the baseline condition, PLV was normalized with the mean and the standard deviation (over time) of PLV of the baseline condition [4,12] as:.

Since PLVz measures the difference from baseline PLV, it is less sensitive to synchrony arising via volume conduction from sources.

####  Classification of phase-locked clusters

**Figure 2:**
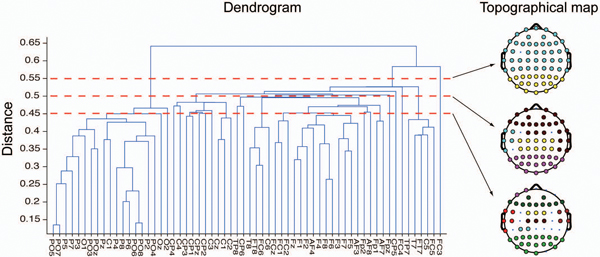
**Sample dendrogram and topographical maps. **Left: an example of a dendrogram as a result of the hierarchical clustering. The x-axis indicates EEG recording electrode sites. The y-axis represents the “distance” between cortical areas. The distance indicates how strongly signals from distant cortical areas are phase-locked (see Methods for details). Note that the geometry of electrodes in the topographical map did not factor into the calculation of the distance. Right: each topographical map is obtained from a corresponding clustering threshold (designated as a red dotted line) in the left dendrogram. Note that the colour itself does not have any meaning. Electrodes marked with “x” indicate clusters that have only one node.

Using PLV or PLVz, we classified cortical areas into phase-locked clusters. To obtain such clusters, we applied the hierarchical clustering to the PLV and PLVz data. The hierarchical clustering sequentially binds up the nearest electrodes based on the “distance” or similarity between cortical signals recorded with electrode *i* and *j* at time *t* defined below. For clustering with PLV, distance was defined as

so that all distance scores are larger than 0. For the clustering with PLVz, negative PLVz scores were reset to 0 before applying the clustering algorithm, because our interest is in significantly enhanced phase-locking. Distance between cortical signals recorded with electrode *i* and *j* was defined as

where PLVz for a given electrode pair and condition at time *t* was subtracted from the maximum score of PLVz across electrode pairs, conditions and time. Note that for determining the distance, the geometry of electrodes in the topographical map did not factor into the calculation in either PLV or PLVz clustering. Additionally, the distance between groups was defined as the minimum distance of all the pair of electrodes between the groups (nearest neighbour). The result of the binding process is expressed as a hierarchical tree or dendrogram (Figure 2, left). Each clustering threshold gives corresponding phase-locked clusters (Figure 2, right) by pruning branches off the bottom of the dendrogram, and assigning all the objects below each cut to a single cluster. Clustering obtained by a higher threshold regime gives weakly phase-locked clusters.

## Results

### The stable component of phase-locked clusters throughout the test time

**Figure 3:**
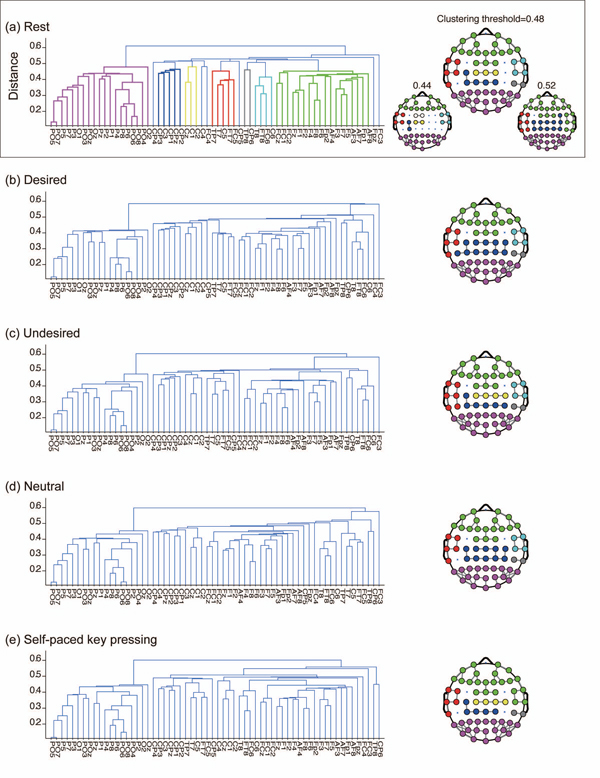
**Time-averaged dendrograms and clusters on a topographical map when the clustering threshold is 0.48** Clustering results with the time-averaged PLV matrix from 750 ms before key press to 750 ms afterwards. In the dendrograms on the left, the y-axis indicates “distance” between electrodes defined as 1-PLV. The topographical maps on the right are obtained when the clustering threshold is set at 0.48 in the dendrograms on the left. PLV links that are greater than 0.52 are superimposed as gray lines for reference. In (a), the colour of the electrodes in the dendrograms on the left corresponds to the colour of the phase-locked cluster on the right. For comparison among conditions, the same cortical regions are kept roughly the same colours.

We investigated phase-locked clusters from time-averaged PLV in order to elucidate the spatial structure of phase-locked clusters. The clustering results are shown in Figure 3 (left) as dendrograms. In the resting state condition, rigid clusters were organized in occipital and frontal areas. These features were also observed in the dendrograms of the other perceptual conditions. This similarity suggests the existence of phase-locked clusters conserved across the perceptual conditions.

**Figure 4:**
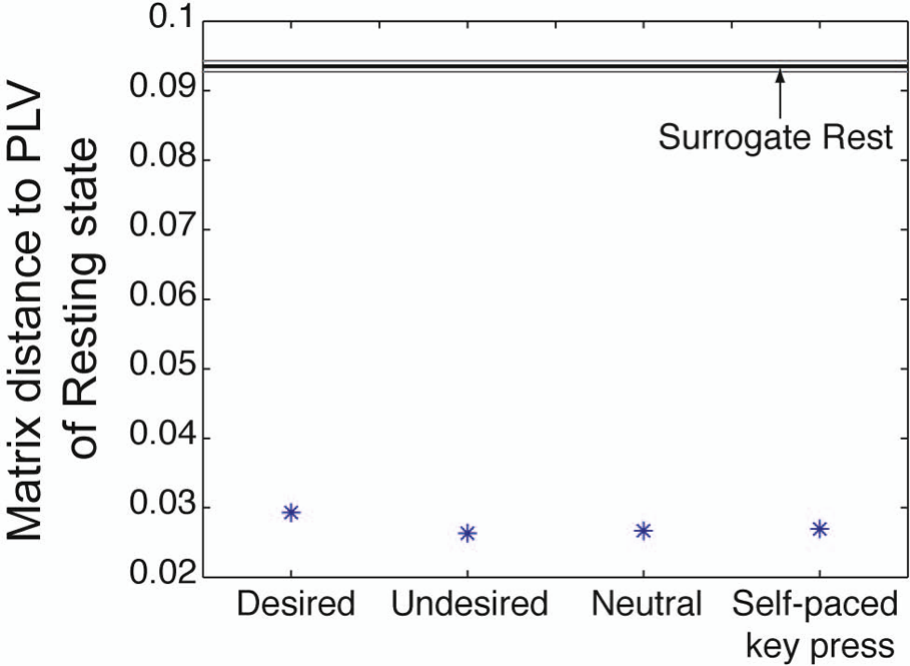
**Matrix distances between the resting state and perceptual conditions** The y-axis indicates the Euclid distance of the PLV matrix of the perceptual conditions to that of the resting state condition. The horizontal black line is the distance of the surrogate resting state condition to the original resting state condition. To obtain the surrogate PLV of the resting state, epochs were randomly chosen for each recording electrode. For each of the 100 surrogate PLVs, the distance to the original PLV of the resting state was obtained. Gray lines represent mean ± 3 SD

To estimate the similarity of the dendrograms, we calculated the Euclid distance between the PLV matrix of the resting state and those of the other perceptual conditions. Smaller Euclid distance indicates the similarity of the matrices and therefore the dendrograms obtained from them. In Figure 4, these distances are compared to the distance between the PLV matrix of the resting state and the surrogate matrix of the resting state. The surrogate PLV matrices were generated by randomly shuffling the EEG signals across epochs and calculating PLVs for this shuffled data as for the original data. As the figure shows, the former distances were far shorter than the latter distances. This result confirms the existence of intrinsic phase locking pattern between signals at distant cortical areas.

To evaluate the significance of phase-locking as a whole pattern of the cortical network, we calculated the Euclid distance between the surrogate matrix and the corresponding original PLV matrix. The resulting distances were: 0.1039±0.7858×10^-3^ (desired), 0.1010±0.8643×10^-3^ (undesired), 0.1027±0.7363×10^-3^ (neutral), 0.0995±0.7852×10^-3^ (self-paced key press), 0.0936±0.7561×10^-3^ (rest) (mean ± 3 SD), all of which were clearly larger than 0. These results indicate that the intrinsic phase-locking pattern is significant compared to the background fluctuation.

In Figure 3 (right), we showed the topographical maps of the phase-locked clusters obtained with the clustering threshold of 0.48. Note that different colours in these figures indicate different clusters but the identity of the colours does not have any meaning. A common feature across the conditions is that the clusters are divided into occipital, parietal and frontal areas. A similar clustering pattern was observed for the range of the threshold from 0.44 to 0.52 (Figure 3(a), right). This indicates that the clustering pattern is not sensitive to the clustering threshold. We also confirmed that the clustering pattern was stable over time. These observations suggest that this pattern is stable across time and conditions. The clustering pattern is comparable to structural connectivity obtained from the diffusion spectrum imaging (Figure 6a in [13]). Although the clustering algorithm adopted in this study is not exactly the same as ours, some features are common to the modules of structural connectivity and the clusters of phase-locking (see Discussion for details).

### Transient change of phase-locked cluster

In the previous section, we observed that phase-locked clusters have a common stable structure across the conditions. In this section, we investigate the transient change of phase-locking from the stable structure. For this purpose, we normalize PLV relative to the time-averaged PLV in the resting state condition (called PLVz. See Methods for details.) By applying the hierarchical clustering to the PLVz, we can extract the transient component of the phase-locked activity that runs on the condition-independent clusters observed in the previous section.

**Figure 5:**
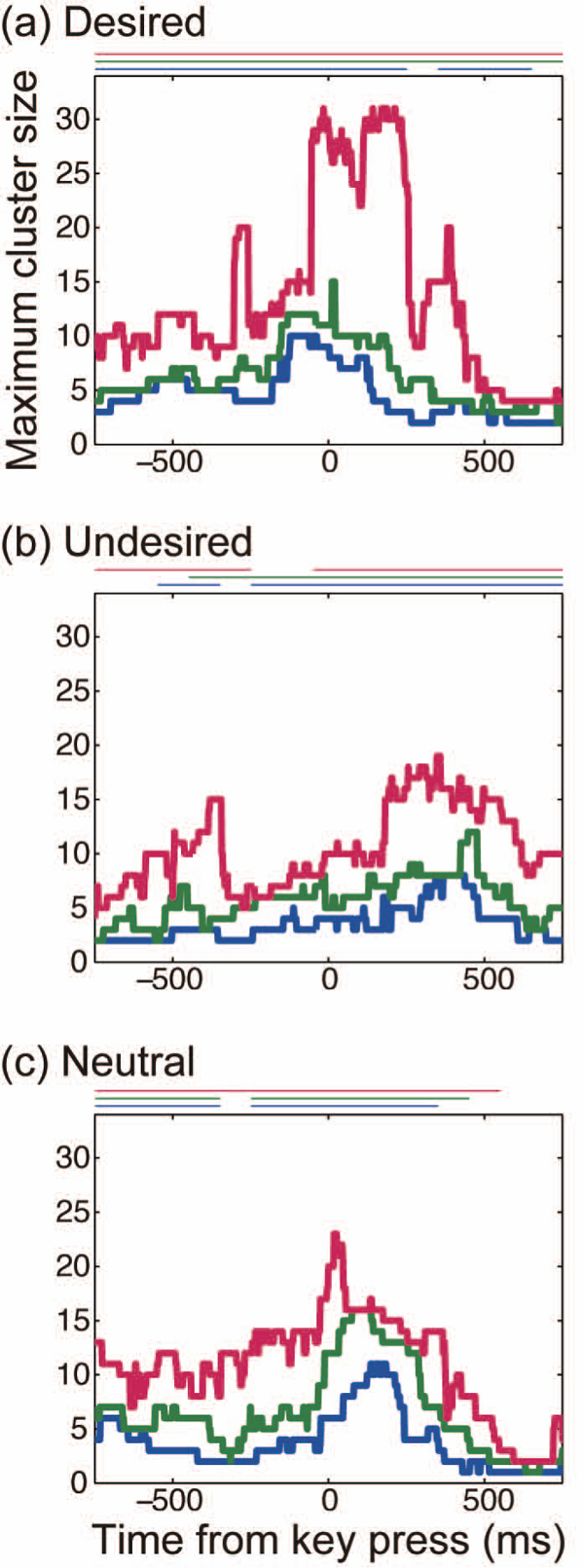
**Time course of maximum cluster size for different clustering thresholds** Time courses of phase-locked clusters obtained from the PLVz matrix. Red, green and blue lines represent the clustering result when the clustering threshold = 7.0, 8.0, and 9.0, respectively in PLVz units. The y-axis indicates the maximum cluster size at each time. Here, the cluster size is defined as the number of recording electrodes that belong to the identical cluster. Horizontal lines above each figure represent the periods during which the maximum cluster size is significantly larger than that in the self-paced key press condition.

Temporal evolutions of the cluster size are displayed in Figure 5, in which clustering thresholds are 7.0, 8.0, and 9.0 (expressed in units of PLVz). In the desired switch at each threshold, the cluster gradually grew to its maximum around the key press time (0 ms). The same tendency was seen in the neutral switch. In the undesired switch, cluster size was maximized at 400 ms. Thus, strength and timing of the modulation of the phase-locked clusters depended on perceptual conditions.

To check whether these changes in cluster size were due to the evoked activity by the key press, we compared the cluster size between a perceptual condition (desired, undesired, neutral) and the self-paced key press condition by applying a permutation test to the time series data of maximal cluster size. We first set time windows of 100 ms and did a random partition to the combined data set of the two conditions within the window (we confirmed that the result of the test was not sensitive to the window size). On this random partition, the average of the maximum cluster size was calculated as a test static. We repeated these procedures for 50000 times and obtained a histogram of the test static. From the histogram and the test static that was actually observed, a p-value was obtained. The total significance level (p < 0.05) was controlled by the number of time windows (i.e. 15 windows). The significant periods (p < 0.05 in total, p < 0.003 for each time window) are displayed in Figure 5 as horizontal lines. Within these periods, it is considered that the changes in the maximum cluster size were not due to the key press, but to perceptual changes. We alternatively performed the permutation test between a perceptual condition and the self-paced key press condition, confirming that the null hypothesis was rejected for even wider periods.

**Figure 6:**
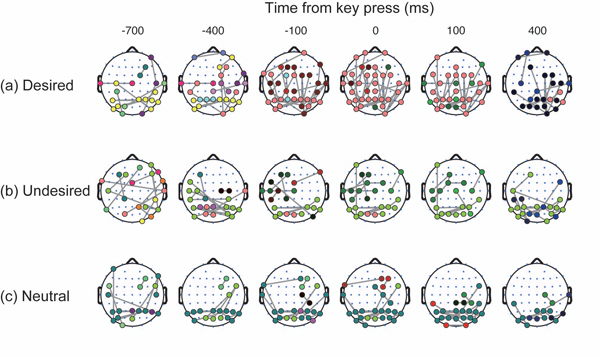
**Time course of phase-locked clusters on topographical maps** Spatio-temporal patterns of clustering results with PLVz at each time section. Topographical maps are obtained when the clustering threshold = 7.0 in units of PLVz. Gray lines represent PLVz links that are greater than 7.0. Note that the same colour in the same condition indicates the identical cluster.

**Figure 7:**
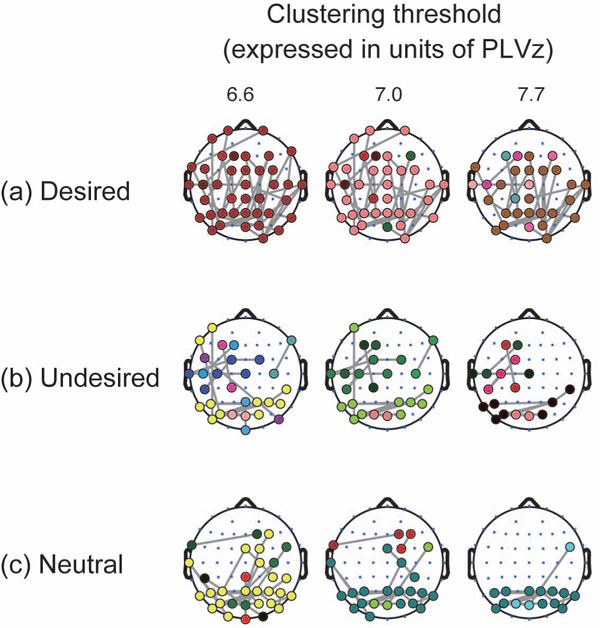
**Phase-locked clusters at different clustering thresholds** Spatial patterns of clustering results with PLVz at the key-press time. Results from different clustering thresholds are laid out in line. Note that colour itself does not have any meaning. The same colour in different threshold conditions does not imply the same cluster.

We subsequently investigated how the modulation of the cluster depends on perceptual conditions from the topographic aspects. Figure 6 shows the spatio-temporal patterns of the clusters of different conditions when the clustering threshold is set at 7.0. In the desired switch, two initially separated clusters at the frontal and occipital areas connected with each other at 0 ms. This integration of the clusters accounts for the maximization of cluster size at the key press time observed in Figure 5(a). In the undesired switch, however, the clusters localized in the occipital and left parietal areas remained divided, even when the cluster size is maximized (at 400 ms). This separation pattern is comparable to the time-averaged clustering pattern (Figure 3, right). Likewise, in the neutral switch, the cluster in the occipital area remained separated from clusters in the other areas. Although there existed a small cluster connecting the occipital and parietal areas during -400 to 0 ms, this cluster and the other cluster in the occipital area did not merge. Instead, the cluster in the occipital area grew to its maximum at 0 ms within the occipital area. We observed this difference of the spatial clustering pattern across the perceptual conditions for the range of the threshold from 6.6 to 7.7 (Figure 7). We thereby conclude that the difference of cluster size among the conditions as observed in Figure 5 is explained by the difference in spatial organization of the clusters shown in Figure 6.

Integrating the temporal and spatial characteristics described above leads to a dynamical nature toward global synchrony, especially in the desired switch. Small clusters that coexist at the beginning gradually merge one after another to organize a large cluster around the key press time. It can be postulated that there would be several possible ways to achieve global phase synchrony. One possibility is growth of only one small core cluster. Another possibility is merging of coexisting clusters. The dynamics observed in the desired switch are more consistent with the latter view than the former.

## Discussion

Previous studies have suggested that frontal-occipital synchrony network is enhanced when top-down selective attention is involved in perceptual switch. Our result on transient modulation of phase-locked clusters is consistent with this notion. In addition, our result suggests that processes towards synchrony are also different depending on desired, undesired and neutral switch. In the top-down attentional conditions (desired and undesired switch), phase-synchrony in occipital and frontal areas was independently enhanced before the perceptual switch. In contrast, in the neutral switch, only occipital areas were enhanced. Subsequently, occipital and frontal areas were transiently connected more strongly when top-down attention succeeded in switching perception (desired switch) than when top-down attention did not succeed (undesired switch).

The relationship between functional network and anatomical structure has been investigated in detail [14-17] while transient changes of this relationship have not been yet clarified. However, brain functions can be adaptive faster than anatomical structures so that the non-stationarity or metastability of functional networks should be critical in the processing of perception, as Bullmore and Sporns pointed out in [18]. Our results suggest that stable functional clusters (Figure 3, right) are transiently connected more strongly for the desired switch than for the other conditions. More theoretical and modelling studies are needed to reveal the dynamical mechanism of the transient change of phase-locking, especially the dynamical clustering.

The dynamical clustering observed in this report may be related to functional clustering observed in globally coupled nonlinear systems [19-26]. In these systems, when multiple identical elements with nonlinear dynamics interact through a mean field or through a random network, the elements differentiate into some clusters. In each cluster, elements oscillate synchronously although elements in different clusters oscillate with different amplitude, frequency, or phase. The idea of this clustering has been extended to cell differentiation [27,28] and pattern dynamics in gas-discharge systems [29] as well as in experiments of electro-chemical oscillations [30-32]. One feature that may be useful in the context of neuroscience is its flexibility in making clusters.

In investigating the stable component of the phase-locked clusters around the time of perceptual switch, we found that the clusters were divided into occipital, parietal, and frontal areas (Figure 3, right). This clustering pattern is comparable to the modules of structural connectivity reported in [13]. In this study, structural connectivity was estimated with the use of the diffusion spectrum imaging in which nerve fiber architecture is mapped from the 3D spectra of tissue water diffusion with magnetic resonance. From the resulting cortical connection matrix, structural modules were identified employing spectral graph partitioning [33]. Hagmann et al. reported that optimal modularity was achieved with six clusters. In particular, four contralaterally matched modules were localized to the frontal and temporo-parietal areas of a single hemisphere. The two remaining modules comprised regions of the bilateral medial cortex, one centred on the posterior cingulate cortex and another centred on the precuneus and pericalcarine cortex. We also applied the same clustering algorithm [33] to our data and confirmed that the resulting phase-locked clusters were closer to the structural connectivity reported in [13] than the cluster obtained from the hierarchical clustering (Figure 3, right). Nevertheless, these clusters had similar structure to the structural connectivity in that the clusters were divided into occipital, parietal and frontal areas. This result is thereby consistent with the previous knowledge obtained from modelling [34,35] and physiological studies [13-16], which reported that functional clusters coincide with the topological community structures. Thus it is natural to expect that via cortico-cortical synaptic connections [36], phase-locking around 4Hz is mediated.

Other than the hierarchical clustering that we used, there have been some clustering algorithms, for example, based on network modularity [14,33] and based on an information-theoretic perspective [37]. In this report, however, we chose a rather simple algorithm, hierarchical clustering, because we were interested in the degree of phase-locking rather than network structure. In addition, our method is suitable for observing clusters that have the same degree of phase-locking over time. In contrast, the criteria for clustering are not fixed in the other methods.

In this report, we characterized the transient process towards attention-enhanced global synchrony as dynamical clustering. It would be interesting to investigate whether the dynamical clustering is observed in the other global synchrony phenomena that are selectively enhanced by perceptual states such as consciousness. We hope our findings will help to reveal the unique process of global synchrony and unveil the dynamical mechanism that constitutes conscious processing.

## Conclusions

Through hierarchical clustering, we investigated functional phase-locked clusters during Necker cube perception. The spatial structure of the clusters was stable across time and conserved across the perceptual conditions. We also observed that these stable clusters were transiently connected around the time of perceptual switch. This transient modulation was stronger in the desired switch than in the other conditions. These observations suggest the characteristic dynamics towards the attention-modulated global synchrony in which small clusters that coexisted at the beginning gradually merge one after another to organize a large cluster around the time of perceptual switch.

## List of abbreviations

EEG: electroencephalogram; EOG: electrooculogram; PLV: phase locking value

## Competing interests

The authors declare that they have no competing interests.

## Authors' contributions

DS performed analysis and drafted the manuscript. KKitajo and YY conceived and designed the experiments. KKitajo ran the experiments. KKitajo, KKaneko, and YY helped to draft the manuscript.
